# IL-33 Receptor-Expressing Regulatory T Cells Are Highly Activated, Th2 Biased and Suppress CD4 T Cell Proliferation through IL-10 and TGFβ Release

**DOI:** 10.1371/journal.pone.0161507

**Published:** 2016-08-22

**Authors:** Julia Siede, Anja Fröhlich, Angeliki Datsi, Ahmed N. Hegazy, Domonkos V. Varga, Vivien Holecska, Hirohisa Saito, Susumu Nakae, Max Löhning

**Affiliations:** 1 Experimental Immunology and Osteoarthritis Research, Department of Rheumatology and Clinical Immunology, Charité-Universitätsmedizin Berlin, Berlin, Germany; 2 Pitzer Laboratory of Osteoarthritis Research, German Rheumatism Research Center (DRFZ), a Leibniz Institute, Berlin, Germany; 3 Translational Gastroenterology Unit, Nuffield Department of Clinical Medicine, Experimental Medicine Division, John Radcliffe Hospital, University of Oxford, Oxford, United Kingdom; 4 The Kennedy Institute of Rheumatology, Nuffield Department of Orthopaedics, Rheumatology and Musculoskeletal Sciences, University of Oxford, Headington, Oxford, United Kingdom; 5 Department of Allergy and Immunology, National Research Institute for Child Health and Development, Tokyo, Japan; 6 Laboratory of Systems Biology, Center for Experimental Medicine and Systems Biology, The Institute of Medical Science, The University of Tokyo, Tokyo, Japan; 7 Precursory Research for Embryonic Science and Technology, Japan Science and Technology Agency, Saitama, Japan; University of Lisbon, PORTUGAL

## Abstract

Immunomodulatory Foxp3^+^ regulatory T cells (Tregs) form a heterogeneous population consisting of subsets with different activation states, migratory properties and suppressive functions. Recently, expression of the IL-33 receptor ST2 was shown on Tregs in inflammatory settings. Here we report that ST2 expression identifies highly activated Tregs in mice even under homeostatic conditions. ST2^+^ Tregs preferentially accumulate at non-lymphoid sites, likely mediated by their high expression of several chemokine receptors facilitating tissue homing. ST2^+^ Tregs exhibit a Th2-biased character, expressing GATA-3 and producing the Th2 cytokines IL-5 and IL-13 –especially in response to IL-33. Yet, IL-33 is dispensable for the generation and maintenance of these cells *in vivo*. Furthermore, ST2^+^ Tregs are superior to ST2^−^ Tregs in suppressing CD4^+^ T cell proliferation *in vitro* independent of IL-33. This higher suppressive capacity is partially mediated by enhanced production and activation of the anti-inflammatory cytokines IL-10 and TGFβ. Thus, ST2 expression identifies a highly activated, strongly suppressive Treg subset preferentially located in non-lymphoid tissues. Here ST2^+^ Tregs may be well positioned to immediately react to IL-33 alarm signals. Their specific properties may render ST2^+^ Tregs useful targets for immunomodulatory therapies.

## Introduction

Regulatory Foxp3^+^ CD4^+^ T cells (Tregs) are key controllers of immune homeostasis. They maintain immune tolerance, thus preventing autoimmunity or excessive inflammation [1, 2]. They are present in almost all tissues, even under homeostatic conditions, and regulate a variety of innate and adaptive immune cells [3, 4]. Various mechanisms mediating Treg functions have been described. These include direct suppression or cytolysis of target cells, repression of APC maturation and function as well as secretion and activation of anti-inflammatory cytokines such as IL-10 and TGFβ [5, 6]. Consequently, Tregs form a heterogeneous population displaying diverse migratory properties and immunomodulatory effects.

A minor fraction of Tregs in the circulation and lymphatic organs exhibits an activated effector/memory T cell phenotype similar to conventional T cells, thus termed effector Tregs. These Tregs are assumed to have encountered antigen more recently and preferentially reside in non-lymphoid tissues (NLT) [7]. Several surface markers distinguishing effector Tregs have been identified so far, including αE integrin (CD103) which marks a subset of highly suppressive, rapidly activated Tregs that preferentially resides in NLT [8–10]. A similar phenotype is observed in KLRG1-expressing Tregs that accumulate in the lung in a model of airway inflammation, accompanied by increased levels of CD44, CD69, CD25, CTLA-4 and a downregulation of CD62L [11, 12]. In general, effector Tregs display classical T cell activation markers, like CD44^hi^ and CD62L^lo^, along with molecules involved in Treg maintenance and function, such as Foxp3, CTLA-4, KLRG1, CD103 and ICOS and are thought to be highly suppressive.

A growing body of evidence suggests that the acquisition of an effector-like phenotype does not mark the end point of Treg differentiation. Instead, further diversification comparable to conventional T cells may occur [13–16]. Notably, the Th2 lineage-specifying transcription factor GATA-3 can be upregulated in Tregs upon encounter of antigen and IL-2 [17, 18]. In Th2 cells, GATA-3 induces transcription of the *Il1rl1* gene, encoding ST2, the receptor for the alarmin IL-33 [19]. Recently, it was shown that ST2 is also expressed on a subset of Tregs in a GATA-3-dependent manner [20]. Additional studies revealed that systemic administration of IL-33 increased the number of total and ST2^+^ Tregs resulting in a delay of graft-versus-host disease and amelioration of colitis [21–23]. Moreover, in a setting of acute inflammation, IL-33 signals are essential for the accumulation of ST2^+^ Tregs in mucosal tissue and the stability of the Treg phenotype [20]. Yet, ST2^+^ Tregs are present in several organs even under homeostatic conditions [24], but their phenotype and suppressive capacity at steady-state remain ill defined.

Here we report that ST2^+^ Tregs are highly activated effector Tregs that preferentially accumulate in NLT. They exhibit a Th2-like phenotype with elevated expression of GATA-3 and production of the Th2 cytokines IL-5 and IL-13, which can be further augmented by IL-33 signals. In line with their effector-like phenotype, ST2^+^ Tregs suppress naïve CD4^+^ T cell proliferation more effectively than their ST2^−^ counterparts–independent of IL-33. Both IL-10 and increased TGFβ production and activation contribute to the suppressive mechanism employed by ST2^+^ Tregs. Finally, we demonstrate that IL-33 is dispensable for the generation, maintenance and tissue accumulation of ST2^+^ Tregs under homeostatic conditions. Taken together, ST2^+^ Tregs form a highly suppressive subset located in prime position to react to inflammatory processes involving the release of IL-33.

## Material and Methods

### Mice

*Il33*^-/-^ [25], *Il1rl1*^-/-^ [26] and WT mice were bred on C57BL/6 background under SPF conditions at the Charité animal facility, Berlin. *B6*.*Foxp3*^*hCD2*^ reporter mice [27] were crossed to *Il10*^*gfp*^ reporter [28] under SPF conditions at animal facilities at the University of Oxford, UK. Experiments were performed at the Charité and DRFZ, Berlin, in accordance with national law for animal protection with permission from the Landesamt für Gesundheit und Soziales (Lageso); permission number T0058/08. Experiments at the University of Oxford were approved by the Clinical medicine AWERB committee at the University of Oxford.

Animals were euthanized by cervical dislocation.

### T cell isolation for *ex vivo* characterization

Blood was drawn from animals before they were euthanized and perfused with PBS. Circulating lymphocytes were isolated by high density centrifugation using Histopaque (Sigma-Aldrich). Lungs were chopped, treated with 0.1U/ml Collagenase D (Roche), homogenized and applied to a Histopaque gradient. Spleen and LN were homogenized and splenic erythrocytes were lysed. Payer’s patches were removed from the small intestine (SI). Colon and SI were cut and incubated in RPMI with 1mM DTT, 5mM EDTA, 5% FCS, homogenized and treated with 0.1U/ml Collagenase D. Single cell suspensions were applied to a 40/70% Percoll gradient.

### Flow cytometric analysis and sorting

Samples were stained with antibodies against CD4 (GK1.5), CD25 (PC61), KLRG1 (2F1), CD103 (2E7), CD62L (MEL-14), CD44 (IM7), LPAM-1/α4β7 (DATK32), CD183/CXCR3 (CXCR3-173), CD194/CCR4 (2G12), CD196/CCR6 (29-2L17), CD197/CCR7 (4B12), CD199/CCR9 (CW-1.2), anti-hCD2 (RPA-2.10) and ST2 (DJ8) in PBS/0.2% BSA/2mM EDTA. ST2 staining was amplified using FASER-Kit-PE or FASER-Kit-APC (Miltenyi Biotec). CTLA-4 (UC10-4B9) staining was performed after fixation. T-bet (4B10), GATA-3 (TWAJ), and Foxp3 (FJK-16S) were stained using the Foxp3 staining buffer set (eBioscience). GATA-3 index depicts the MFI ratio of GATA-3 and its respective isotype (eB149/10H5) staining.

Samples were acquired on FACSCanto II (BD Biosciences) and analyzed using FlowJo (FlowJo) or FCAP array software (BD).

### T cell cultures and suppression assays

CD4^+^ T cells from spleen and LN were enriched by magnetic cell separation (MACS, Miltenyi Biotec). CD4^+^ CD25^+^ ST2^+^ or CD4^+^ CD25^+^ ST2^−^ T cells (Tregs) and CD4^+^ CD62L^+^ CD44^lo^ (Tresp) were purified by FACS. For Treg stimulation, 96-well flat-bottom plates were coated with 3μg/ml anti-CD3 (145-2C11) and 6μg/ml anti-CD28 (37.51) antibodies. 2x10^4^ to 1x10^5^ Tregs were resuspended in 140–200μl of RPMI/10% FCS supplemented with 40ng/ml IL-2 with or without 30ng/ml IL-33. After 60-70h, supernatants were collected and cytokines were quantified by cytometric bead array (BD Pharmingen).

For suppression assays, Tresp were labeled with CellTrace Violet or CFSE (Invitrogen). APCs were isolated from splenocytes by MACS using biotinylated anti-CD19 (1D3), anti-CD11b (M1/70) and anti-CD11c (HL3) antibodies and anti-biotin microbeads (Miltenyi Biotec). IL-10 signaling was blocked by pre-incubation of Tresp with 50μg/ml anti-CD210/IL-10R (1B1.3a). TGFβ signaling was blocked using 10μM TGFβRI inhibitor in DMSO (SB431542, Merck Millipore) directly in culture. Control cells were treated with DMSO alone. The Treg:Tresp ratio was varied as indicated; twice as much APCs and 5μg/ml anti-CD3 antibody were added. Proliferation of Tresp was analyzed on day 4 by flow cytometry. The division index indicates the average number of divisions that a cell in the starting population has undergone. The relative division index was normalized to the corresponding untreated population.

### mRNA isolation and quantification

RNA was isolated using the miRNeasy mini kit (Qiagen). Complementary DNA was synthesized using the Taqman Reverse Transcription Reagents (Life Technologies). Quantitative RT-PCR reactions were performed in duplicates using SYBR Select Master Mix (Life Technologies), respective primers (S1 Table) on a QuantStudio 7 real-time PCR system (Life Technologies). Data was normalized to *Hprt* endogenous control.

All reagents and kits were used at manufacturer’s recommendation, if not stated otherwise.

### Statistical analysis

Two groups were compared with two-tailed unpaired Student’s t test (GraphPad Prism 5.02); * p ≤ 0.05; ** p ≤ 0.01; *** p ≤ 0.001; non-significant (ns) p > 0.05.

## Results

### ST2^+^ Tregs arise independently of IL-33 signals

**Fig 1:**
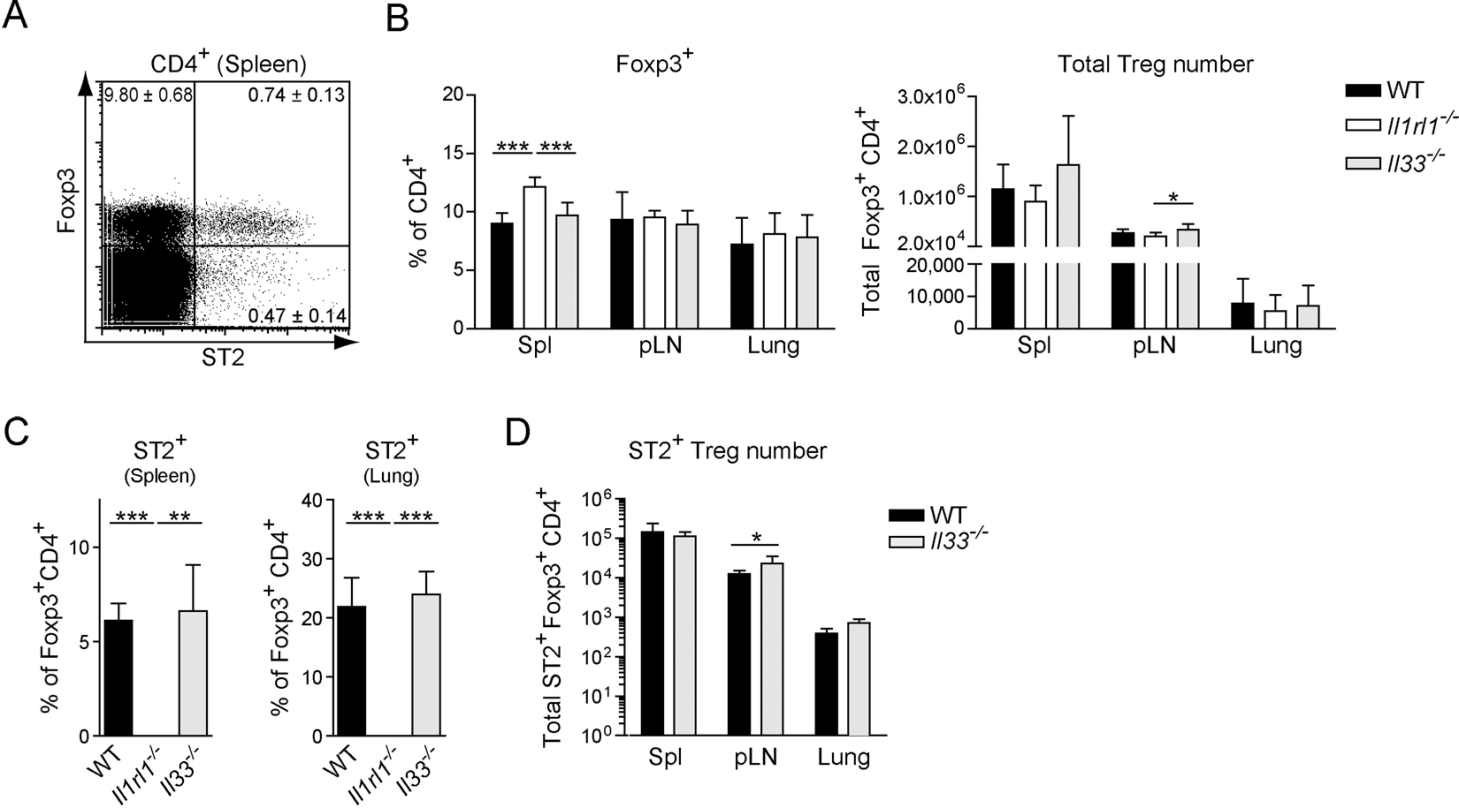
ST2^+^ Tregs arise independently of IL-33 signals. Phenotype of Tregs in naive WT, *Il1rl1*^*-/-*^ and *Il33*^*-/-*^ mice: *(A)* ST2 and Foxp3 expression by splenic CD4^+^ T cells of one representative naive WT mouse; quadrant numbers indicate the average frequency ± SD in 4 mice. *(B)* Frequencies and total numbers of FoxP3^+^ Tregs in spleen (Spl), peripheral lymph nodes (pLN) and lung. *(C)* Frequency of ST2 expression in Tregs of spleen and lung. *(D)* Total number of ST2^+^ Tregs in spleen, pLN and lung. Fig *2A*, *2C* and *2D*: Data are representative of at least 2 independent experiments. Fig *2B*: Pooled data from 2 independent experiments, each with 4 mice per genotype. Bar graphs show the mean ± SD of at least 4 individual mice. Significance was tested using unpaired Student’s t test. Asterisks indicate significance; all others non-significant. * p ≤ 0.05; ** p ≤ 0.01; *** p ≤ 0.001.

First, we wanted to assess whether IL-33 signaling via the ST2 receptor is necessary for the development and maintenance of ST2^+^ Tregs present under homeostatic conditions. Therefore we isolated splenocytes from naive WT mice, and analyzed the frequency of ST2^+^ and ST2^−^ Foxp3^+^ CD4^+^ T cells (Fig 1A and S1A Fig). As reported previously a subpopulation of Tregs expressed the receptor for IL-33. Next, we determined the frequency and number of total Tregs in WT, ST2-deficient (*Il1rl1*^*-/-*^) and *Il33*^*-/-*^ mice. We found that both the frequency and number of Tregs were largely comparable in the spleen, peripheral lymph nodes (pLN) and lung of these mice (Fig 1B). Although a significantly elevated frequency of splenic Tregs was detected in *Il1rl1*^*-/-*^ mice, this did not translate into significant differences in the total splenic Treg numbers between the three genotypes. Likewise, the frequencies of ST2^+^ Tregs in the spleen and lung of WT and *Il33*^*-/-*^ mice were similar (Fig 1C), as were the numbers of ST2^+^ Tregs in spleen, pLN and lung (Fig 1D). Moreover, no differences were observed in the functional capacity of WT and *Il1rl1*^*-/-*^ Tregs to suppress WT responder T cell proliferation in the presence or absence of IL-33 signals (S2 Fig). Taken together, these data show that IL-33 signaling is dispensable for the generation, maintenance and suppressive function of Tregs, including ST2^+^ Tregs, at steady-state.

### ST2^+^ Tregs preferentially home outside of secondary lymphoid organs

**Fig 2:**
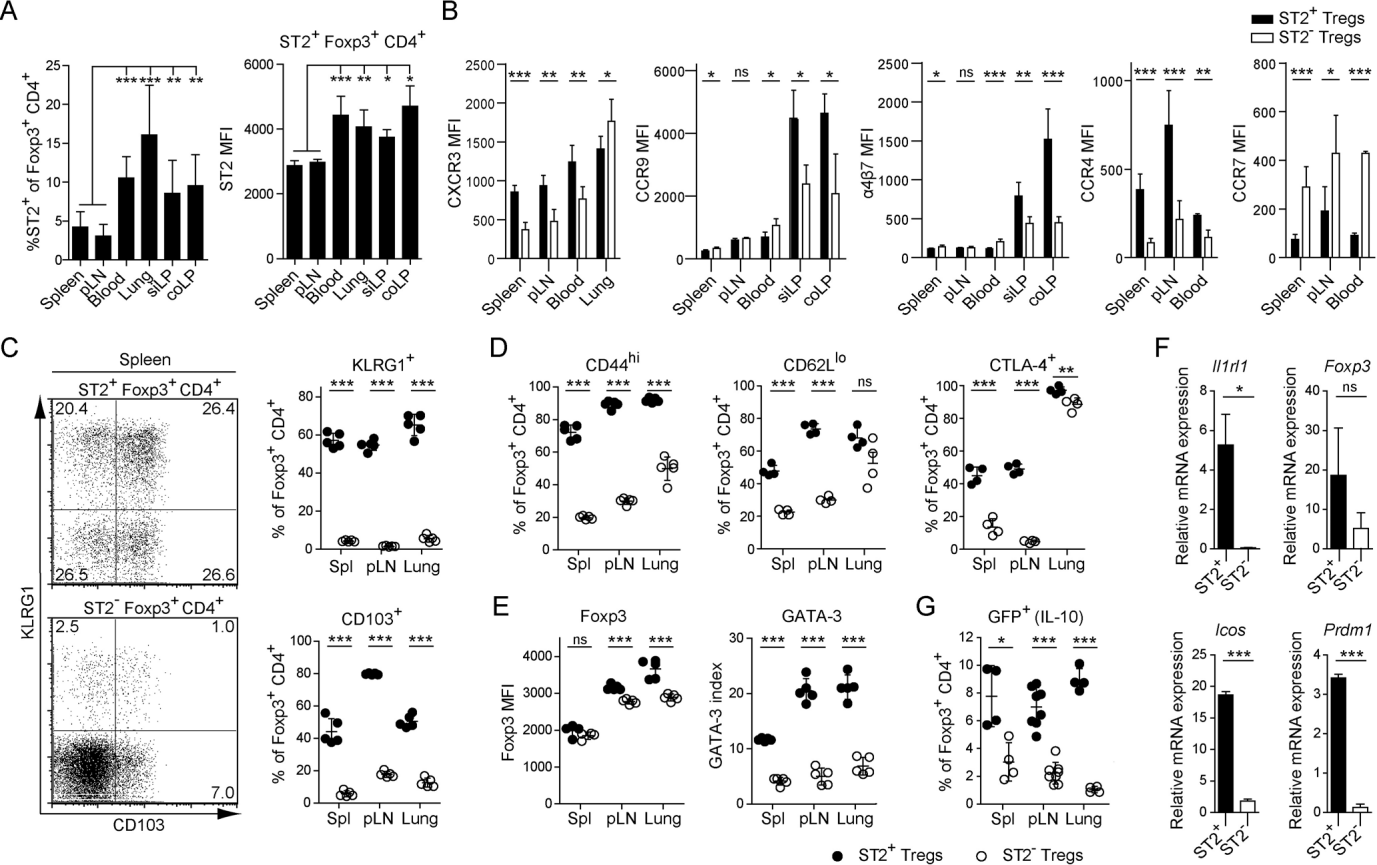
ST2^+^ Tregs preferentially home outside of secondary lymphoid organs and exhibit a highly activated phenotype. Flow cytometric analysis of the phenotype and frequency of WT ST2^+^ and ST2^−^ Foxp3^+^ Tregs in spleen, pLN, blood, lung, lamina propria of the small intestine (siLP) and colon (coLP): *(A)* Frequency of ST2^+^ Tregs *(left)* and MFI of the ST2 staining on the ST2^+^ Treg fraction *(right)*. *(B)* MFI of chemokine receptor and α4β7 staining on ST2^+^ and ST2^−^ Tregs. *(C)* KLRG1 and CD103 expression in ST2^+^ *(top)* and ST2^−^ *(bottom)* Tregs from spleen; quantified frequencies from indicated organs *(right)*. *(D)* Frequency of CD44^hi^, CD62L^lo^ and CTLA-4^+^ T cells within ST2^+^ and ST2^−^ Treg populations. *(E)* MFI of the Foxp3 staining *(left)* and geometric mean index of GATA-3 *(right)* in ST2^+^ and ST2^−^ Tregs. *(F)* Quantification of mRNA expression of the indicated genes from FACS-sorted ST2^+^ and ST2^−^ CD25^+^ Tregs from spleen and pLN *ex vivo*. mRNA expression normalized to *Hprt* endogenous control. *(G)* Frequency of ST2^+^ and ST2^−^ Tregs with IL-10 production capability as detected by GFP expression from *B6*.*Foxp3*^*hCD2*^ *xIl10*^*gfp*^ reporter mice. Fig *2A*: Data are representative of at least 2 independent experiments. Bar graphs show the mean ± SD of at least 5 biological replicates. Fig *2B*: pooled data from 2 independent experiments with 3–5 biological replicates each. Bar graphs show the mean ± SD. Fig *2C–2E* and *2G*: Data are representative of at least 2 independent experiments. Scatter plots depict one mouse as individual dot with mean ± SD. Fig *2F*: pooled data from 2 independent experiments. Significance was tested using unpaired Student’s t test. * p ≤ 0.05; ** p ≤ 0.01; *** p ≤ 0.001; non-significant (ns) p > 0.05.

As distinct Treg subsets exhibit different migratory properties [29], we compared the frequency of ST2^+^ Tregs in lymphoid (spleen, pLN) and non-lymphoid organs (lung, lamina propria of the small intestine (siLP) and colon (coLP)) of naive WT mice. While ST2^+^ Tregs were present in all organs, the highest frequency was found in non-lymphoid tissue (NLT), especially the lung (Fig 2A left). In addition, the ST2-expressing Tregs at these sites expressed more ST2 on a per cell basis (Fig 2A right), suggesting a preferential accumulation or development of highly ST2^+^ Tregs in the NLT. In agreement with these data, we found that ST2^+^ Tregs have an increased homing capacity to NLT based on their chemokine receptor expression (Fig 2B): First, CXCR3, involved in Treg recruitment to inflammatory sites [30], was significantly higher expressed on ST2^+^ than ST2^−^ Tregs in all organs analyzed but the lung, suggesting an increased potential of ST2^+^ Tregs to home to those organs. However, expression was highest in the lung hinting towards a general requirement for lung-homing Tregs to express CXCR3. Second, CCR9 and α4β7 integrin are strong promoters of lymphocytic migration to the intestinal lamina propria [31]. Their expression was significantly higher on ST2^+^ than ST2^−^ Tregs found in the gut. Third, CCR4 has been implicated in enhanced migration of Tregs to the skin and lung to control local inflammation [10]. We found significantly higher CCR4 expression on ST2^+^ Tregs, suggesting preferential migration to these sites. Last, CCR7, a homing marker for secondary lymphoid organs [32, 33], was significantly lower expressed on ST2^+^ than ST2^−^ Tregs. Taken together, these results indicate that ST2^+^ Tregs accumulate in NLT, presumably facilitated by their distinct chemokine receptor and integrin expression pattern.

### ST2^+^ Tregs display a highly activated phenotype

Next, we analyzed the expression of molecules associated with Treg function and activation on ST2^+^ and ST2^−^ Treg subsets. Both KLRG1 and CD103 have been described to be expressed on highly suppressive Tregs with the capacity to home to inflamed tissues [8–10]. Around 70% of splenic ST2^+^ Tregs expressed either one or both of these markers compared to only about 10% in the ST2^−^ Treg compartment. A similar distribution was observed in the pLN and lung (Fig 2C). These data suggest that the ST2^+^ Treg subset is largely comprised of highly differentiated and suppressive Tregs. To provide further evidence for the activated phenotype of ST2^+^ Tregs, two classical activation-associated surface molecules were assessed. While CD44 expression on Tregs is correlated with increased suppressive capacity [34], the role of CD62L in Treg functionality is controversially discussed [35–37]. We found that ST2^+^ Tregs expressed high levels of CD44 and low levels of CD62L. Additionally, CTLA-4 is crucial for the suppressive function of Tregs [38]. Indeed, the frequency of CTLA-4^+^ Tregs was clearly increased in the ST2^+^ compartment, especially within lymphoid tissues (Fig 2D). Moreover, we analyzed the expression of transcription factors in the ST2^+^ and ST2^−^ Treg subsets. Both the Treg-key regulator Foxp3 as well as the Th2-associated transcription factor GATA-3 were elevated in ST2^+^ Tregs (Fig 2E and S3A Fig). In contrast, expression of T-bet, which can be induced in Tregs upon Th1-differentiating antigen encounter [13], was not altered (S3B Fig). Although specific to ST2^+^ Tregs, we found the expression of all of the aforementioned molecules to be independent of IL-33 (S3C and S3D Fig).

At the mRNA level we could confirm a higher expression of *Il1rl1* (ST2) and *Foxp3* in ST2^+^ Tregs (Fig 2F). Furthermore, the expression of two molecules associated with a highly activated and suppressive Treg phenotype, *Icos* and *Prdm1* (Blimp1) [39, 40], was significantly increased in the ST2^+^ compared to the ST2^−^ Treg subset. Next, we assessed the production of the anti-inflammatory cytokine IL-10, whose expression in Tregs under homeostatic conditions is dependent on Blimp1 [40] and has been shown to confer Treg function [41]. In all organs, ST2^+^ Tregs displayed an increased capability to produce IL-10 when compared with ST2^−^ Tregs, with greatest differences detectable in the lung (Fig 2G). Overall, these data suggest that the ST2^+^ Treg subset combines various characteristics of highly activated, differentiated and suppressive Tregs.

### ST2^+^ Tregs exhibit increased suppressive capacity *in vitro*

**Fig 3:**
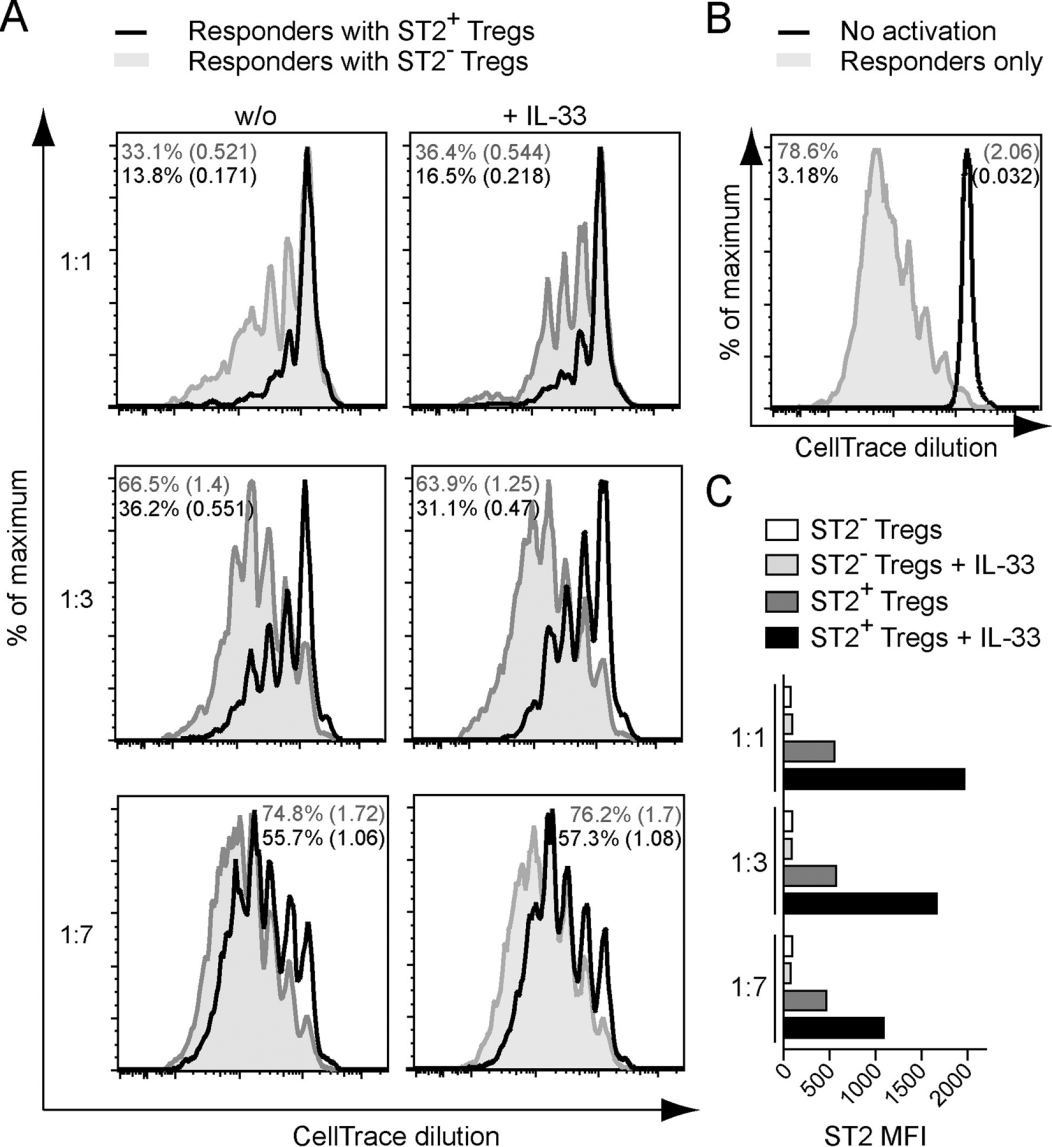
ST2^+^ Tregs suppress CD4^+^ T cell proliferation more effectively than ST2^−^ Tregs *in vitro*. *(A)* Proliferation profiles of CellTrace-labelled WT CD25^-^ CD62L^hi^ CD4^+^ responder T cells (Tresp) co-cultured with WT ST2^+^ (black) and ST2^−^ (grey) CD25^+^ Tregs during an *in vitro* suppression assay at day 4 of culture. T cells were stimulated by APCs and anti-CD3 antibody with *(right column)* or without *(left column)* the addition of recombinant IL-33. Treg:Tresp ratios are indicated *(left)*. Percentage of divided cells and the division index (number in brackets) are shown in each histogram in the respective color. *(B)* Proliferation profile of Tresp cultured under the same conditions as in 3A but without Tregs, either with (grey) or without (black) the addition of anti-CD3 antibody. *(C)* MFI of the ST2 staining on all Tregs recovered from the cultures described in 3A. Data are representative of 2–3 independent experiments.

In light of the highly activated and differentiated nature of ST2^+^ Tregs, we investigated their suppressive effect on T cell proliferation in *in vitro* suppression assays. ST2^+^ and ST2^−^ Tregs were isolated from spleen and pLN by FACS (S1B Fig) and separately co-cultured with different ratios of naive CD4^+^ responder T cells (Tresp). While both Treg subsets suppressed Tresp proliferation at all ratios tested, ST2^+^ Tregs were considerably more suppressive (Fig 3A and 3B). However, the addition of IL-33 had no beneficial effect on their suppressive capacity, but resulted in a selective increase of ST2 expression on ST2^+^ Tregs (Fig 3C).

Taken together, these results demonstrate that ST2^+^ Tregs strongly suppress CD4^+^ responder T cell proliferation. This property appears to be independent of their ability to receive and process IL-33 signals through the ST2 receptor.

### ST2^+^ Tregs express TGFβ and the Th2 cytokines IL-5, IL-13 and IL-10

**Fig 4:**
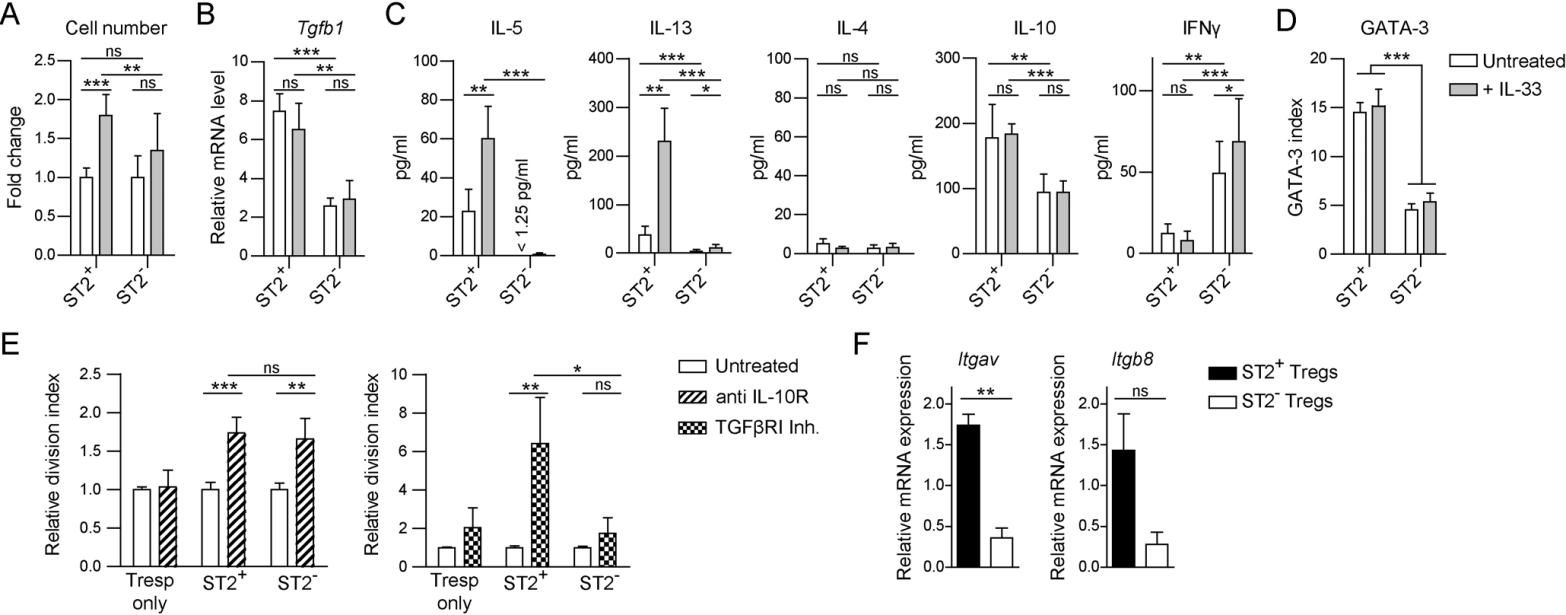
ST2^+^ Tregs express Th2 cytokines and suppress CD4^+^ T cell proliferation via IL-10 and TGFβ. *(A-D)* ST2^+^ and ST2^−^ Tregs from spleen and lymph nodes of WT mice activated *in vitro* by plate-bound anti-CD3/anti-CD28 antibodies in the presence of IL-2 with or without recombinant IL-33 for 60–70 hours: *(A)* Fold change in the number of viable Tregs upon IL-33 treatment. *(B) Tgfb1* mRNA expression normalized to *Hprt* endogenous control. *(C)* Cytokine concentration in the supernatants as determined by cytometric bead array. *(D)* Geometric mean index of GATA-3 in stable ST2^+^ and ST2^−^ Tregs at the end of culture. *(E) In vitro* suppression assay with ST2^+^ and ST2^−^ Tregs as described in Fig 3 (Treg:Tresp ratio 1:5) with addition of blocking anti-IL-10R antibody or TGFβRI inhibitor. The relative division index indicates the fold increase in division of Tresp upon treatment. Division index of untreated Tresp was set to 1 in each group. *(F)* Quantification of mRNA expression of the indicated genes from sorted ST2^+^ and ST2^−^ CD25^+^ Tregs *ex vivo*. mRNA expression normalized to *Hprt* endogenous control. Fig *4A*–*4C*, *4E* and *4F*, data pooled from 2–3 independent experiments each performed with 2 replicates per condition. Fig *4D* is representative of 2 independent experiments with at least 2 replicates per condition each. Bar graphs show the mean ± SD. Significance was tested using unpaired Student’s t test. * p ≤ 0.05; ** p ≤ 0.01; *** p ≤ 0.001; non-significant (ns) p > 0.05.

Although IL-33 did not influence the suppressive capacity of ST2^+^ Tregs *in vitro*, it still increased the amount of ST2 expressed on their cell surface. To assess whether other functions are affected by the capacity of ST2^+^ Tregs to sense IL-33, we stimulated ST2^+^ and ST2^−^ Tregs *in vitro* in the presence or absence of this cytokine. We observed that the number of living cells was highest when ST2^+^ Tregs were cultured in the presence of IL-33 (Fig 4A). Thus, IL-33 affects either their proliferation or survival. Moreover, ST2^+^ Tregs produced more TGFβ, IL-5, IL-13 and IL-10 at the mRNA and protein level than their ST2^−^ counterparts, and the production of the Th2-associated cytokines IL-5 and IL-13 was vastly increased by IL-33 (Fig 4B and 4C, S4 Fig). GATA-3 expression was also significantly higher in ST2^+^ than ST2^−^ Tregs but unaffected by IL-33 signaling (Fig 4D). In contrast, IFNγ secretion was significantly lower by ST2^+^ than ST2^−^ Tregs. Almost no IL-4 was detectable in the supernatants of stimulated ST2^+^ and ST2^−^ Tregs with only minor differences between the groups. Hence, we conclude that IL-33 positively influences the expansion of ST2^+^ Tregs and promotes the production of the Th2 cytokines IL-5 and IL-13.

### ST2^+^ Tregs suppress responder T cells via IL-10 and TGFβ release and activation

Both IL-10 and TGFβ are thought to at least partially mediate the suppressive function of Tregs [42]. Since ST2^+^ Tregs produce significantly higher amounts of these cytokines than their ST2^−^ counterparts, we addressed if these cytokines contribute to the increased suppressive capacity of ST2^+^ Tregs. Therefore, we blocked IL-10 or TGFβ signaling during *in vitro* suppression assays (Fig 4E). The blockade of IL-10 signaling by antibodies targeting the IL-10 receptor increased the proliferation of Tresp to a similar extent in the presence of ST2^+^ and ST2^−^ Tregs, hinting at similar contribution of IL-10 to the suppressive mechanisms of both Treg subsets despite differences in the secreted amount. In contrast, the application of a TGFβ receptor I inhibitor [43] resulted in enhanced proliferation of Tresp cultured in the presence of ST2^+^ Tregs, whereas Tresp alone or cultured with ST2^-^ Tregs were only marginally affected. This finding suggests a major contribution of TGFβ signaling to the suppressive mechanism of ST2^+^ but not ST2^-^ Tregs. In fact, not only the production of total TGFβ was higher in ST2^+^ Tregs but also the expression of integrin αvβ8 (*Itgav* and *Itgb8*) associated with the activation of latent TGFβ [44] (Fig 4F). Taken together, these data demonstrate that ST2^+^ Tregs employ a mechanism based on IL-10 and increased TGFβ production and activation to at least partially mediate their highly suppressive function.

## Discussion

Recently, the expression of the IL-33 receptor ST2 was found on a subset of regulatory T cells [20–22, 24, 45]. However, their phenotype and tissue distribution under homeostatic conditions as well as the suppressive mechanism employed by these cells are still poorly understood. Here we report that ST2^+^ Tregs display a Th2-biased effector Treg phenotype and strongly suppress naïve CD4^+^ T cell proliferation; an effect partially mediated by the anti-inflammatory cytokines IL-10 and TGFβ.

We observed that at steady-state, ST2^+^ Tregs preferentially accumulated at non-lymphoid tissues, such as the lung, the lamina propria of the small intestine and colon as well as the circulation. These findings are in agreement with previous data obtained in inflammatory settings that show an accumulation of ST2^+^ Tregs in the colon [20], the lung and especially the visceral adipose tissue (VAT) [24, 46]. IL-33 is constitutively expressed in the nucleus of epithelial and endothelial cells and released upon necrotic cell death [47, 48]. However, under homeostatic conditions IL-33 release is very limited. Thus, our data demonstrate that signaling via the ST2 receptor is dispensable for the generation, maintenance and tissue accumulation of ST2^+^ Tregs. Notably, VAT Tregs form an exception. This specialized, self-contained Treg subset, which also expresses the transcription factor PPARγ associated with adipocyte differentiation [49], is highly dependent on the IL-33/ST2 axis for their stability and function in the VAT [24, 46, 50].

Regarding ST2^+^ Treg tissue migration, we identified chemokine receptors and integrins associated with NLT homing to be particularly highly expressed in the ST2^+^ Treg compartment, including CXCR3, CCR4, CCR6, CCR9, αE (CD103) and α4β7 integrin. Migration along the respective chemokine gradients could contribute to the positioning and retention of ST2^+^ Tregs at mucosal surfaces already under homeostatic conditions, leaving them in prime position to react to IL-33 danger signals. Low-level release of IL-33 due to constant minor cell damage may contribute to the augmented ST2 expression per cell observed at barrier tissues.

We and others [20, 22, 24] demonstrated that ST2^+^ Tregs belong to a subset of highly activated effector Tregs based on their accumulation at NLT and expression of typical T cell activation markers and molecules involved in Treg maintenance and function. Recently, a subset of effector Tregs expressing the B cell-associated transcription factor Blimp1 was characterized [24, 40]. The authors report that Blimp1 is essential for the production of IL-10 by effector Tregs. Indeed, we found high expression of *Prdm1* (Blimp1) in the ST2^+^ Treg compartment, paired with an increased IL-10 production capability *in vitro* and *in vivo*. Moreover, transcriptome analysis of Blimp1^+^ and Blimp1^-^ Tregs revealed a strong enrichment for ST2 in the Blimp1^+^ Treg subset [24]. Overall, these findings suggest a considerable overlap between the ST2^+^ and Blimp1^+^ effector Treg populations.

The Th2 lineage-defining transcription factor GATA-3 is a known inducer of ST2 expression in Tregs [20]. In line with previous reports [46], we detected particularly high levels of GATA-3 in the ST2^+^ Treg compartment. Furthermore, these cells also secreted increased amounts of Th2-associated cytokines IL-5 and IL-13. In contrast to IL-4 which is produced neither by ST2^+^ nor ST2^-^ Tregs, GATA-3 has been reported to directly bind to the proximal promoters of IL-5 and IL-13 resulting in their expression [51–53]. Additionally the expression of these cytokines can be further augmented by IL-33. However, we did not detect significant changes in GATA-3 expression upon IL-33 signals implying a mere supportive role of ST2 signaling on GATA-3 function. In contrast, *in vivo* administration of IL-33 not only expands Tregs but also increases GATA-3 expression in the ST2^+^ Treg compartment [22], but such a setting is more complex and secondary effects may contribute to the GATA-3 upregulation. Although IL-33 signals significantly expanded or promoted the survival of ST2^+^ Tregs *in vitro*, the increased amounts of the Th2 cytokines IL-5 and IL-13 released by these cells were not only a result of this expansion, but also of increased cytokine transcription. First reports indicate that Th2 cytokine production by human Tregs results in an anti-inflammatory phenotype of alternatively activated macrophages [54]. However, more studies are necessary to determine the full functional consequences of Th2 cytokine secretion by Tregs.

In accordance with their effector-like phenotype [8, 9, 11], ST2^+^ Tregs were superior in suppressing naïve CD4^+^ T cell proliferation compared to their ST2^−^ counterparts. Yet, the comparison of ST2^+^ and ST2^−^ Tregs expanded *in vivo* by IL-33 injection has revealed only insignificant differences in suppressive capacity between the groups [21, 22]. Severe alterations in the hematopoietic compartment upon IL-33 administration [55] including a potential upregulation of ST2 on expanding non-effector Tregs may be the underlying cause of this discrepancy. In line with previous data [20], we observed a similar suppressive capacity of ST2-deficient and WT Tregs, either due to the low abundance of ST2^+^ effector Tregs within the WT Treg compartment or a similar effector Treg frequency in *Il1rl1*^*-/-*^ mice. Furthermore, the addition of IL-33 had no effect on the suppressive capacities of ST2^+^ and ST2^-^ Tregs, despite an expansion of ST2^+^ Tregs. Such suppressive inertia to IL-33 signals might be due to Tregs exerting their most suppressive effects immediately after stimulation, whereas the cell number only gradually increases. Thus, ST2 signaling seems dispensable for the high suppressive function of ST2^+^ Tregs derived from secondary lymphoid organs. However, in NLT-derived ST2^+^ Tregs which express ST2 at a higher per cell amount, IL-33 signaling might be able to enhance their suppressive capacity.

Notably, IL-33 has recently been associated with Treg-mediated wound healing in a number of different tissues [56, 57]. Injury-induced release of IL-33 from the parenchyma resulted in the release of tissue-protective epidermal growth factor ligand amphiregulin from ST2^+^ Tregs [56]. Thus, ST2^+^ Tregs might present dual functionality towards limiting local tissue inflammation: on the one hand by strongly suppressing pro-inflammatory T cell responses and on the other hand by directly triggering tissue repair processes in an IL-33-dependent manner.

A variety of mechanisms have been proposed to mediate Treg suppressive function: (1) direct target killing or suppression via IL-2 deprivation, granzyme B secretion and CD39/73-mediated ATP reduction; (2) repression of APC maturation and function via CTLA-4 and Lag3 signaling; and (3) secretion and activation of anti-inflammatory cytokines IL-10, TGFβ and IL-35 [5, 6]. Indeed, we detected high expression of CTLA-4, IL-10 and TGFβ in the ST2^+^ Treg subset, while others have already identified high levels of Lag3 and CD39 [22]. Furthermore, we provide evidence that both IL-10 and to a greater extend TGFβ contributed to the suppressive capacity of ST2^+^ Tregs. High TGFβ and integrin αvβ8 expression was detected in ST2^+^ Tregs indicating an advantage of ST2^+^ Tregs to produce and activate latent TGFβ [44]. In agreement with the unresponsiveness of ST2^+^ Tregs to IL-33 concerning their suppressive capacity, IL-10 and TGFβ secretion also remained unaffected. Additionally, absence of APCs from the suppression assay system did not alter the suppressive capacity of ST2^+^ Tregs (data not shown) suggesting that under these conditions, ST2^+^ Tregs directly suppress their target cells.

However, the role of IL-10 and TGFβ in suppressing Tresp proliferation *in vitro* remains controversially discussed, with some reports claiming a contribution of these cytokines to the suppressive mechanism of Tregs [58, 59] and others not [60–62]. Yet, the amount of IL-10 and TGFβ produced by the studied Treg populations might strongly influence the observed effect of these cytokines on suppression. As already mentioned, ST2^+^ effector Tregs produce higher amounts of IL-10 and TGFβ compared with their ST2^-^ counterparts. Thus, blockade of IL-10 and TGFβ signaling can exert a greater effect on ST2^+^ Treg-mediated suppression, which is readily detected in *in vitro* suppression assays. However, the reduction in suppression upon IL-10R blockade was comparable between ST2^+^ and ST2^-^ Tregs, despite greater IL-10 production by ST2^+^ Tregs. This observation might be explained by an incomplete IL-10R blockade or a generally minor contribution of IL-10 to Treg-mediated suppression *in vitro*.

In summary, we demonstrate that ST2 expression identifies a highly activated, strongly suppressive Treg subset located in non-lymphoid tissues. The phenotype and function of these cells overlaps in many aspects with previously described effector Treg subsets. However, ST2^+^ Tregs stand out from these populations as they feature a Th2-biased phenotype already under homeostatic conditions. Additionally, these cells employ a mechanism based on IL-10 and increased TGFβ production and activation to mediate their enhanced suppressive function. Due to their specific capabilities, ST2^+^ Tregs may be suitable for targeted immunomodulatory therapies, e.g. to alleviate allergies or autoimmunity.

## Supporting Information

S1 Fig Gating strategies for ST2^+^ Treg analysis and sorting. *(A)* Gating strategy used for *ex vivo* analysis of ST2^+^ Tregs. Representative FACS plots show spleens of WT and *Il1rl1*^*-/-*^ mice. *(B)* Gating strategy used for flowcytometric isolation of ST2^+^ and ST2^-^ Tregs from spleen and pLN (top row). Exemplary counterstaining of CD25 and FoxP3 (bottom row). Data are representative of at least 2 independent experiments.
(TIF)

S2 Fig Comparable suppressive capacity of WT and *Il1rl1*^*-/-*^ Tregs. *In vitro* suppression assay using WT (black line) or *Il1rl1*^*-/-*^ (dotted line) Tregs and *Il33*^*-/-*^ APCs with or without the addition of recombinant IL-33. Proliferation profiles of WT responder T cells (Tresp) shown at day 4 of culture. Treg:Tresp ratio was 1:1. The division index is indicated in each histogram in the respective color. Proliferation of responder T cells without Tregs is shown in grey. Data are representative of 2 independent experiments, each performed with 2 replicates per condition.
(TIF)

S3 Fig Transcription factor and activation marker expression on ST2^+^ and ST2^−^ Tregs. *(A)* Exemplary staining of GATA-3 in splenic ST2^+^ (full line) and ST2^-^ (dotted line) Tregs. Isotype control for GATA-3 is depicted in gray. *(B)* Histogram of T-bet expression by splenic ST2^+^ (black line) and ST2^−^ Tregs (grey) from naive WT mice. T-bet expression by the endogenous T-bet^hi^ CD4^+^ population is depicted as dotted line. *(C)* Frequency of KLRG1, CD103, CTLA-4 and CD44 expressing ST2^+^ and ST2^−^ Tregs in the spleen, pLN and lung of WT and *Il33*^*-/-*^ mice *ex vivo*. *(D)* MFI of GATA-3 in ST2^+^ Tregs of WT and *Il33*^*-/-*^ mice *ex vivo*. Data are representative of at least 2 independent experiments, each performed with 4 replicates per condition. *S3C* Fig: Bar graphs show the mean ± SD. *S3B* Fig: Scatter plots depict one mouse as individual dot with mean ± SD. Significance was tested using unpaired Student’s t test. * p ≤ 0.05; non-significant (ns) p > 0.05.
(TIF)

S4 Fig Cytokine quantity detected in the supernatants of stimulated ST2^+^ and ST2^−^ Tregs is reflected at the mRNA level. Th2-related cytokine mRNA quantified in 70h *in vitro* stimulated ST2^+^ and ST2^−^ CD25^+^ Tregs by pate-bound anti-CD3/anti-CD28 antibodies in the presence of IL-2 with or without IL-33. mRNA expression normalized to *Hprt* endogenous control. Where possible, fold change in regards to untreated ST2^−^ Tregs is displayed. n.d.: non-detectable. Data pooled from 2 independent experiments each performed with 2–4 replicates per condition. Bar graphs show the mean ± SD. Significance was tested using unpaired Student’s t test. * p ≤ 0.05; ** p ≤ 0.01; *** p ≤ 0.001; non-significant (ns) p > 0.05.
(TIF)

S1 Table Murine qPCR primers. (DOCX)

## Abbreviations

coLP: colonic lamina propria
LN: lymph nodes
NLT: non-lymphoid tissues
pLN: peripheral lymph nodes
SI: small intestine
siLP: small intestinal lamina propria
Spl: spleen
Treg: regulatory T cell
Tresp: responder T cells
VAT: visceral adipose tissue
FACS: fluorescence activated cell sorting
