# Supplementary figures and images for: IL-33 Receptor-Expressing Regulatory T Cells Are Highly Activated, Th2 Biased and Suppress CD4 T Cell Proliferation through IL-10 and TGFβ Release
Source: PLoS One. 2016 Aug 22;11(8):e0161507. doi: 10.1371/journal.pone.0161507 (PMC4993514; doi:10.1371/journal.pone.0161507)

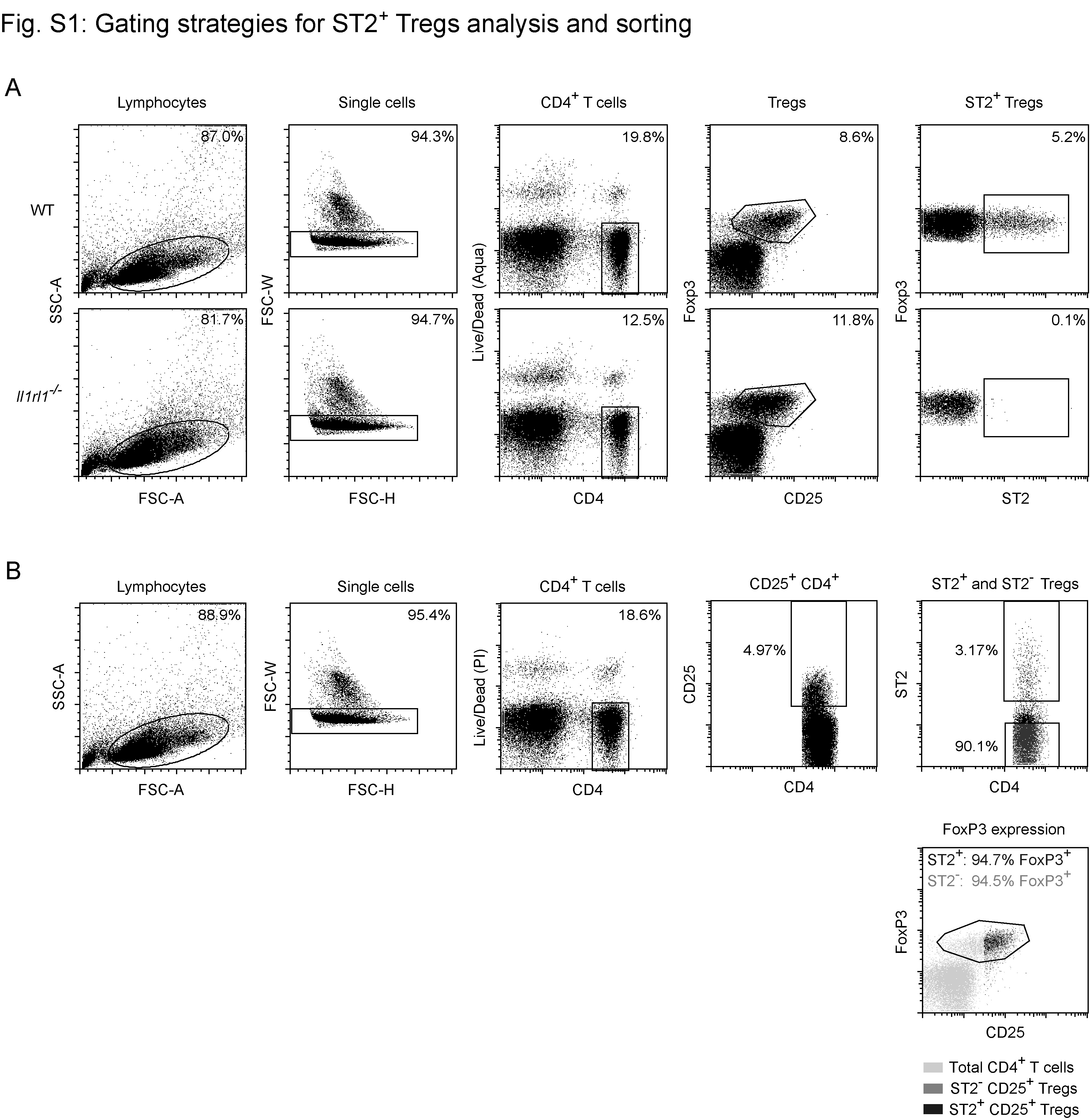

Supplement: S1 Fig — (A) Gating strategy used for ex vivo analysis of ST2+ Tregs. Representative FACS plots show spleens of WT and Il1rl1-/- mice. (B) Gating strategy used for flowcytometric isolation of ST2+ and ST2- Tregs from spleen and pLN (top row). Exemplary counterstaining of CD25 and FoxP3 (bottom row). Data are representative of at least 2 independent experiments. (TIF) [file pone.0161507.s001.tif]

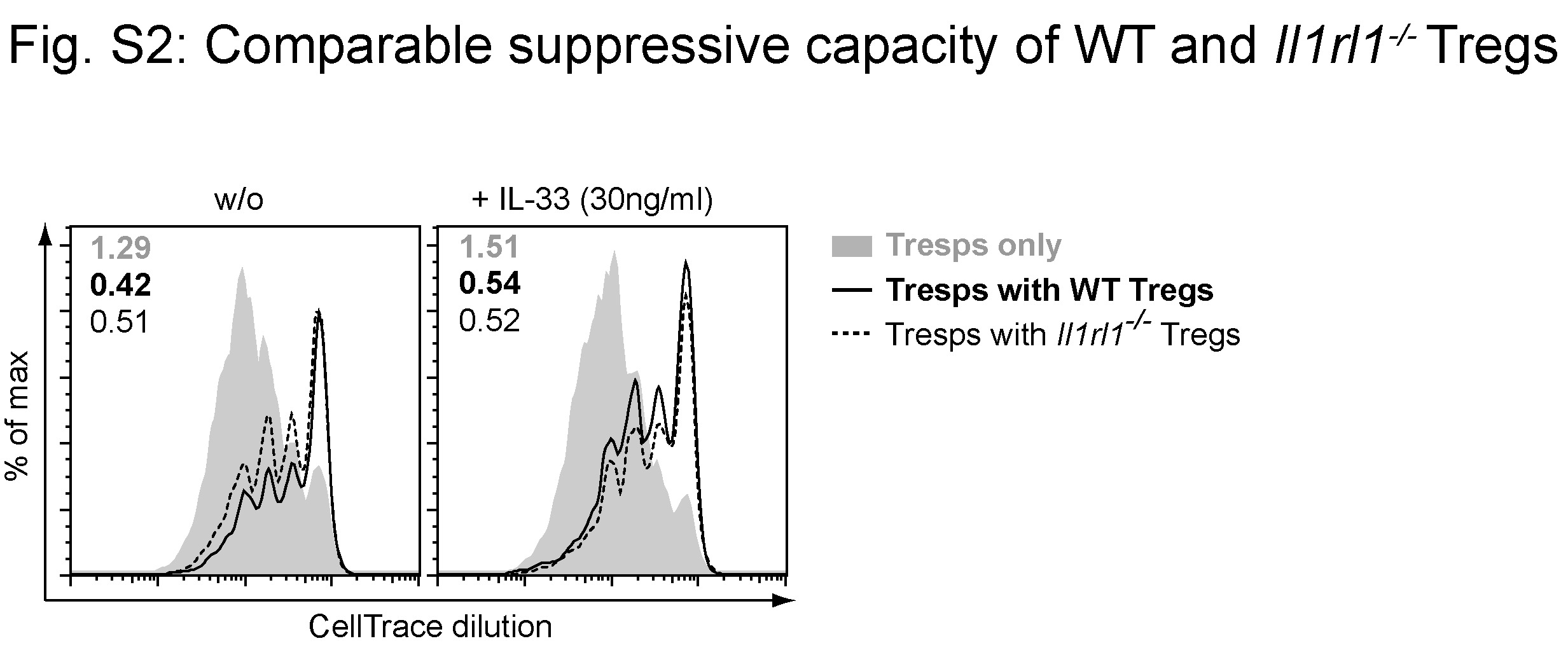

Supplement: S2 Fig — In vitro suppression assay using WT (black line) or Il1rl1-/- (dotted line) Tregs and Il33-/- APCs with or without the addition of recombinant IL-33. Proliferation profiles of WT responder T cells (Tresp) shown at day 4 of culture. Treg:Tresp ratio was 1:1. The division index is indicated in each histogram in the respective color. Proliferation of responder T cells without Tregs is shown in grey. Data are representative of 2 independent experiments, each performed with 2 replicates per condition. (TIF) [file pone.0161507.s002.tif]

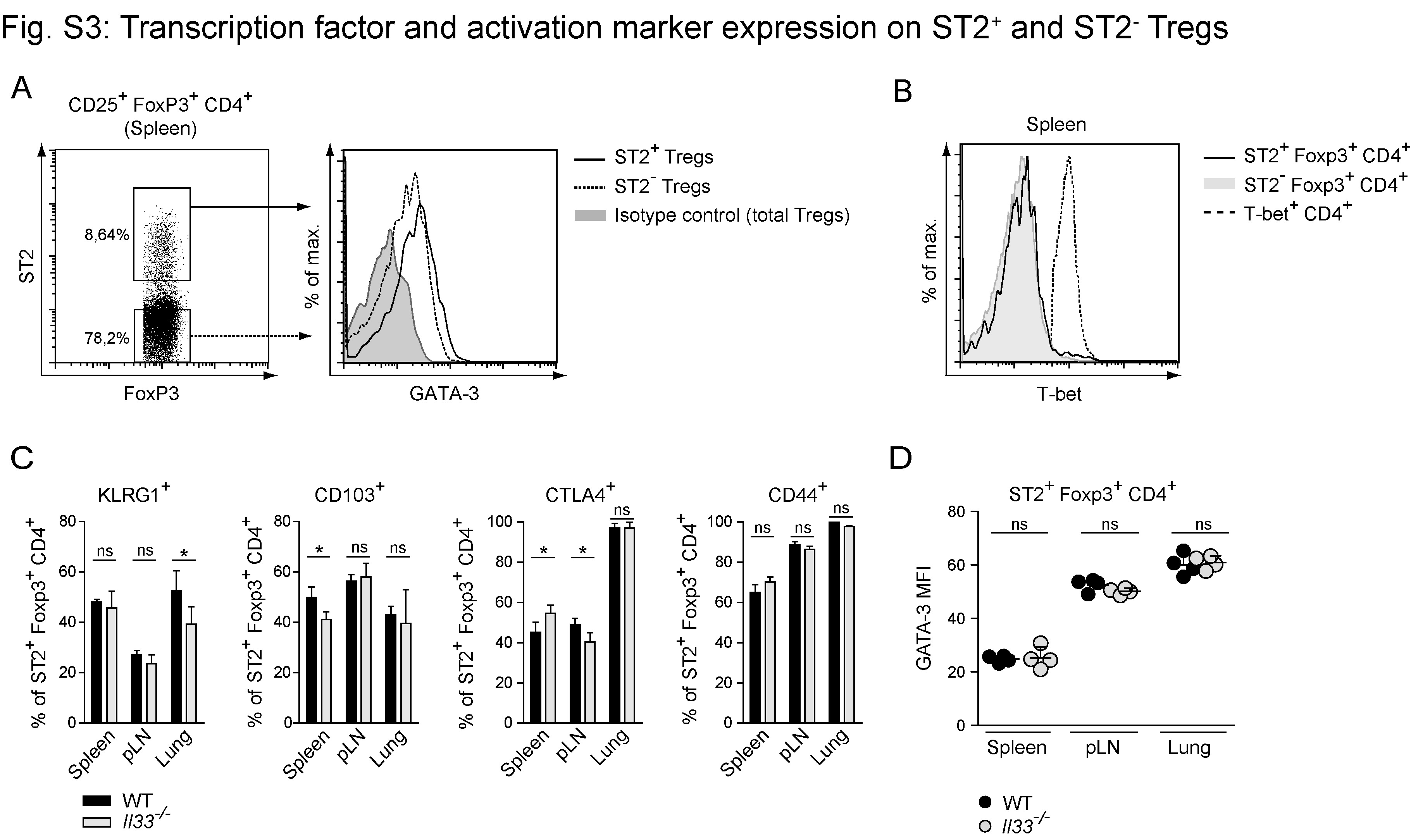

Supplement: S3 Fig — (A) Exemplary staining of GATA-3 in splenic ST2+ (full line) and ST2- (dotted line) Tregs. Isotype control for GATA-3 is depicted in gray. (B) Histogram of T-bet expression by splenic ST2+ (black line) and ST2− Tregs (grey) from naive WT mice. T-bet expression by the endogenous T-bethi CD4+ population is depicted as dotted line. (C) Frequency of KLRG1, CD103, CTLA-4 and CD44 expressing ST2+ and ST2− Tregs in the spleen, pLN and lung of WT and Il33-/- mice ex vivo. (D) MFI of GATA-3 in ST2+ Tregs of WT and Il33-/- mice ex vivo. Data are representative of at least 2 independent experiments, each performed with 4 replicates per condition. S3C Fig: Bar graphs show the mean ± SD. S3B Fig: Scatter plots depict one mouse as individual dot with mean ± SD. Significance was tested using unpaired Student’s t test. * p ≤ 0.05; non-significant (ns) p > 0.05. (TIF) [file pone.0161507.s003.tif]

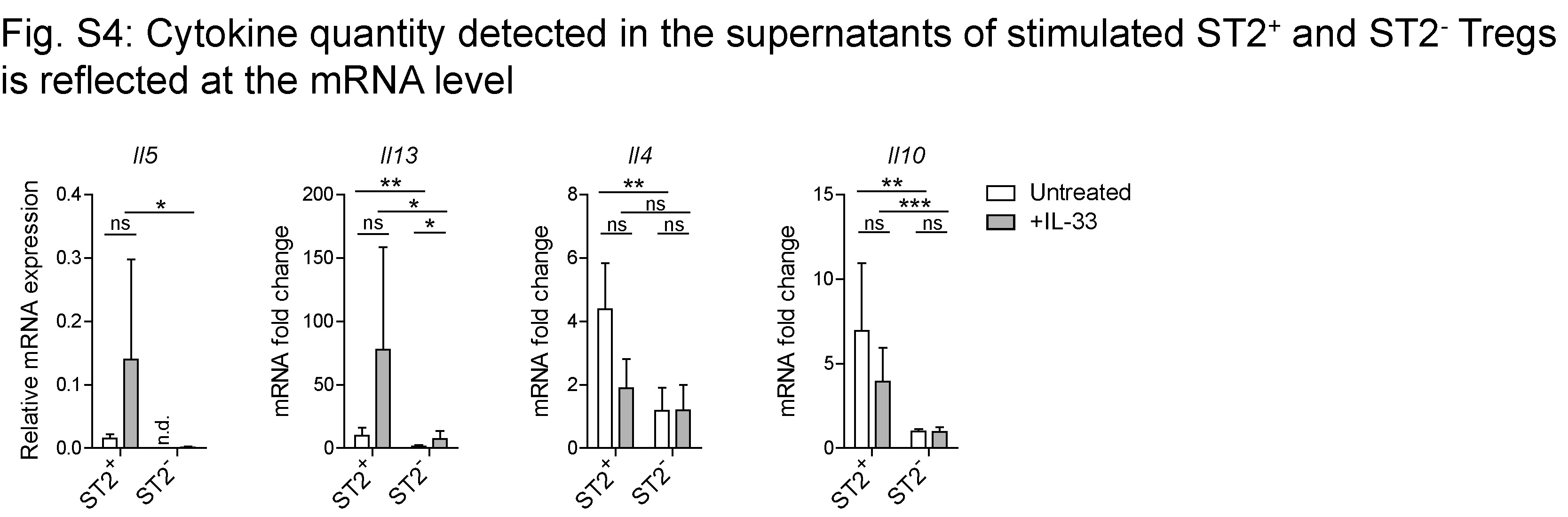

Supplement: S4 Fig — Th2-related cytokine mRNA quantified in 70h in vitro stimulated ST2+ and ST2− CD25+ Tregs by pate-bound anti-CD3/anti-CD28 antibodies in the presence of IL-2 with or without IL-33. mRNA expression normalized to Hprt endogenous control. Where possible, fold change in regards to untreated ST2− Tregs is displayed. n.d.: non-detectable. Data pooled from 2 independent experiments each performed with 2–4 replicates per condition. Bar graphs show the mean ± SD. Significance was tested using unpaired Student’s t test. * p ≤ 0.05; ** p ≤ 0.01; *** p ≤ 0.001; non-significant (ns) p > 0.05. (TIF) [file pone.0161507.s004.tif]
