# Supplementary material for: IL-33 Receptor-Expressing Regulatory T Cells Are Highly Activated, Th2 Biased and Suppress CD4 T Cell Proliferation through IL-10 and TGFβ Release
Source: PLoS One. 2016 Aug 22;11(8):e0161507. doi: 10.1371/journal.pone.0161507 (PMC4993514; doi:10.1371/journal.pone.0161507)
Supplement: S1 Table — (DOCX) [file pone.0161507.s005.docx]

**Table S1: murine qPCR primers**

|  | Forward primer | Reverse primer |
| --- | --- | --- |
| ***Foxp3*** | GCG AAA GTG GCA GAG AGG TA | TCC AAG TCT CGT CTG AAG GC |
| ***Hprt*** | CAT AAC CTG GTT CAT CAT CGC | TCC TCC TCA GAC CGC TTT T |
| ***Icos*** | CGG ATC CAG TGT GCA TGA CC | AGC TTA TGA GGT CAC ACC TGC |
| ***Il4*** | GAG AGG GGA CGC CAT GCA C | GAA GCA CCT TGG AAG CCC TA |
| ***Il5*** | AGC AAT GAG ACG ATG AGG CTT | CCC CCA CGG ACA GTT TGA TT |
| ***Il10*** | GTA GAA GTG ATG CCC CAG GC | GGGGAG AAA TCG ATG ACA GC |
| ***Il13*** | AGA CCA GAC TCC CCT GTG CA | TGG GTC CTG TAG ATG GCA TTG |
| ***Ilrl1*** | CGT GTC CAA CAA TTG ACC TG | CAA GTA GGA CCT GTG TGC CC |
| ***Itgav*** | GGT CGC CTA TCT TCG GGA TG | TGA ACT GGT TCA GGA TGG GC |
| ***Itgb8*** | GGA CTG GGC CAA AGT GAA CA | GAA CAC ACC ATC CGC ATT CC |
| ***Prdm1*** | GAC GGG GGT ACT TCT GTT CA | GGC ATT CTT GGGACC TGT GT |
| ***Tgfb1*** | GTC ACT GGA GTT GTA CGG CA | GGG CTG ATC CCG TTG ATT TC |
